# Poly[di­methyl­ammonium [(μ_2_-benzene-1,2-di­carboxyl­ato-κ^2^ *O* ^1^:*O* ^3^)[μ_2_-3-(pyri­din-4-yl)-1*H*-pyrazol-1-ido-κ^2^ *N* ^1^:*N* ^3^]cuprate(II)]]

**DOI:** 10.1107/S1600536813016334

**Published:** 2013-06-19

**Authors:** Liu Na

**Affiliations:** a Department of Chemistry, Hengshui University, Heng Shui 053000, People’s Republic of China

## Abstract

In the title complex, {(C_2_H_8_N)[Cu(C_8_H_4_O_4_)(C_8_H_6_N_3_)]}_*n*_, there are two Cu^II^ cations (each located on a centre of inversion), one benzene-1,2-di­carboxyl­ate dianion, one 3-(pyridin-4-yl)-1*H*-pyrazol-1-ide anion and one di­methyl­ammonium cation in the asymmetric unit. The di­methyl­ammonium cation was highly disordered and was treated with the SQUEEZE routine in *PLATON* [Spek (2009 ▶). *Acta Cryst.* D**65**, 148–155]; the crystallographic data takes into account the presence of the cation. Each Cu^II^ cation exhibits a square-planar coordination geometry. A benzene-1,2-di­carboxyl­ate dianion bridges two Cu^II^ cations, building a linear chain along [001]. The chains are connected by 3-(pyridin-4-yl)-1*H*-pyrazol-1-ide anions, constructing a layer parallel to (101). The layers are assembled into a three-dimensional supra­molecular network through C—H⋯π inter­actions.

## Related literature

For background to complexes derived from 4-(1*H*-pyrazol-3-yl)pyridine, see: Davies *et al.* (2005 ▶); Tan *et al.* (2011 ▶); For background to complexes derived from benzene-1,2-di­carb­oxy­lic acid, see: Guo (2010 ▶); Yan *et al.* (2012 ▶).
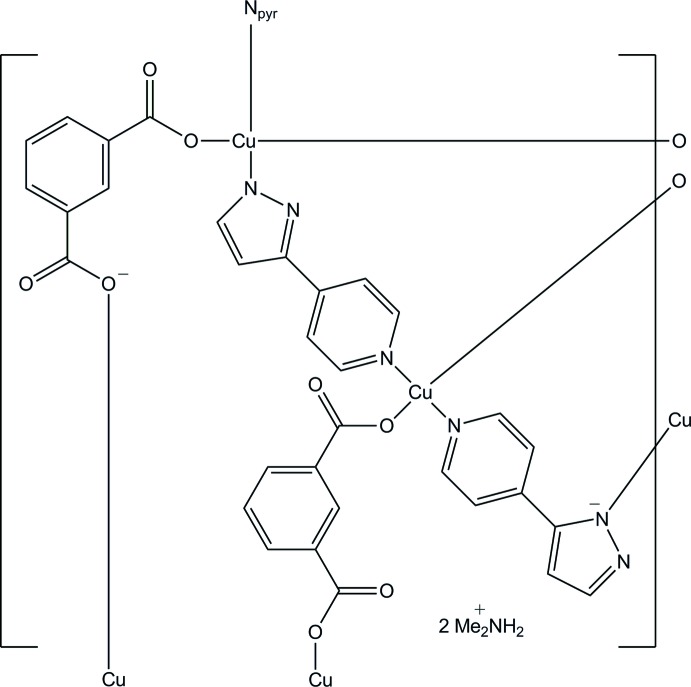


## Experimental

### 

#### Crystal data

(C_2_H_8_N)[Cu(C_8_H_4_O_4_)(C_8_H_6_N_3_)]
*M* *_r_* = 417.91
Triclinic, 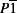

*a* = 8.0978 (16) Å
*b* = 9.7244 (19) Å
*c* = 11.694 (2) Å
α = 89.26 (3)°
β = 89.12 (3)°
γ = 89.64 (3)°
*V* = 920.7 (3) Å^3^
*Z* = 2
Mo *K*α radiation
μ = 1.22 mm^−1^
*T* = 293 K
0.24 × 0.22 × 0.21 mm

#### Data collection

Rigaku SCXmini diffractometer
Absorption correction: multi-scan (*ABSCOR*; Higashi, 1995 ▶) *T*_min_ = 0.759, *T*_max_ = 0.784
8051 measured reflections
3236 independent reflections
2486 reflections with *I* > 2σ(*I*)
*R*_int_ = 0.044

#### Refinement

*R*[*F* ^2^ > 2σ(*F* ^2^)] = 0.046
*wR*(*F* ^2^) = 0.124
*S* = 1.06
3236 reflections
220 parameters
H-atom parameters constrained
Δρ_max_ = 0.50 e Å^−3^
Δρ_min_ = −0.30 e Å^−3^

### 

Data collection: *CrystalClear* (Rigaku, 2005 ▶); cell refinement: *CrystalClear*; data reduction: *CrystalClear*; program(s) used to solve structure: *SHELXS97* (Sheldrick, 2008 ▶); program(s) used to refine structure: *SHELXL97* (Sheldrick, 2008 ▶); molecular graphics: *ORTEPII* (Johnson, 1976 ▶) and *DIAMOND* (Brandenburg, 1999 ▶); software used to prepare material for publication: *SHELXL97*.

## Supplementary Material

Crystal structure: contains datablock(s) I, global. DOI: 10.1107/S1600536813016334/tk5232sup1.cif

Structure factors: contains datablock(s) I. DOI: 10.1107/S1600536813016334/tk5232Isup2.hkl

Additional supplementary materials:  crystallographic information; 3D view; checkCIF report

## Figures and Tables

**Table 1:** Hydrogen-bond geometry (Å, °) *Cg*1 is the centroid of the N2,N3,C9–C11 ring.

*D*—H⋯*A*	*D*—H	H⋯*A*	*D*⋯*A*	*D*—H⋯*A*
C7—H7⋯*Cg*1^i^	0.93	2.85	3.698 (5)	152

Symmetry code: (i) 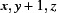.

**Fig. 1.**
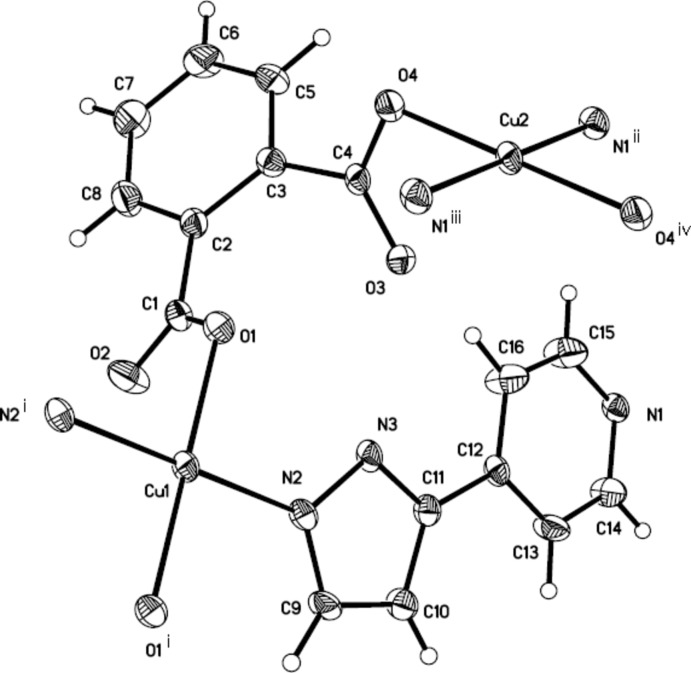
The structure of the title compound, showing the atomic numbering scheme with 30% probability displacement ellipsoids; disordered cation omitted. [Symmetry codes: (i) 2 - x, 1 - y, 1 - z, (ii) 1 + x, y, z, (iii) 1 - x, 1 - y, - z, (iv) 2 - x, 1 - y, - z.]

**Fig. 2.**
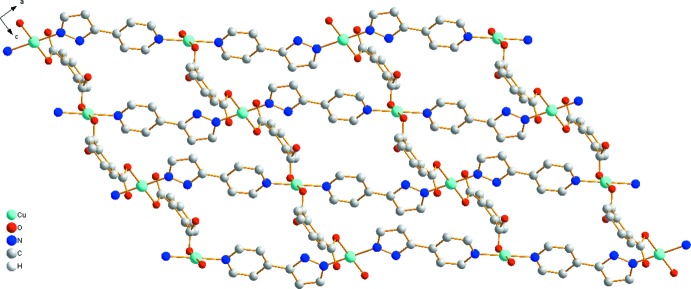
A view of two dimensional layer of title compound; disordered cation omitted.

**Fig. 3.**
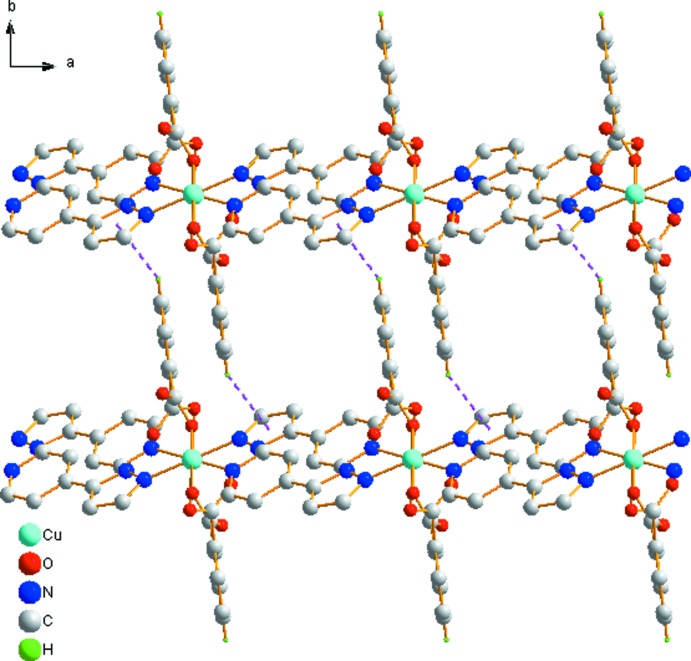
A view of the three-dimensional constructed by C—H···π; disordered cation omitted.

## supplementary crystallographic information

### Comment

The immense research in coordination polymers is due to their potential applications and the diversity in topological architectures. The choice of organic linker is crucial for constructing coordination polymers. Polycarboxylate linkers and *N*-containing ligands are popular for building novel architectures. Among various polycarboxylate linkers, benzene-1,2-dicarboxylic acid (1,2-H_2_bdc) exhibits rich coordination modes to metal centers owing to its rigidity and polycarboxylate groups (Guo, 2010; Yan *et al.*, 2012). On the other hand, among the *N*-containing ligands, 4-(1*H*-pyrazol-3-yl)pyridine (*L*) processes molecular recognition sites for C—H···π and π-π interactions to form interesting supramolecular structures (Davies *et al.*, 2005; Tan *et al.*, 2011). Herein, we report the synthesis and structure of a novel Cu(II) coordination polymer with 1,2-H_2_bdc and *L*.

As illustrated in Fig. 1, there are two types of Cu^II^ cations in the asymmetric unit. Cu(1) exhibits a square planar coordination sphere, defined by two N atoms from two pyrazole rings and two O atoms from two different carboxylate ligands. Cu(2) also shows a square planar coordination geometry with two N atoms and two O atoms. However, two N atoms come from the pyridine rings from *L*. Benzene-1,2-dicarboxylic acids adopt only a single µ_2_-(η^1^,η^1^) bis-monodentate coordination mode connecting two Cu^II^ ions and leads to a one-dimensional linear chain. The chains are connected by *L* to construct a two-dimensional layer (Fig. 2). The layers are further self-assembled into a three-dimensional supramolecular network through C—H···π interactions (Fig. 3 & Table 1).

### Experimental

A mixture of copper nitrate (0.2 mmol), 4-(1*H*-pyrazol-3-yl)pyridine (0.2 mmol), benzene-1,2-dicarboxylic acid (0.2 mmol) were dissolved in a DMAC/Ethanol/H_2_O solvent mixture (5 ml, v:v:v = 1:1:1), and placed in a capped vial (10 ml), which was heated to 373 K for three days and then cooled to room temperature. The crystals obtained were washed with water and dried in air. Element analysis, calculated for C_18_H_18_CuN_4_O_4_: C 51.69, H 4.31, N 13.40%; found: C 51.74, H 4.24, N 13.44%.

### Refinement

Carbon-bound H atoms were placed at calculated positions and were treated as riding on the parent atoms with C—H = 0.93 Å, and with *U*_iso_(H) = 1.2 *U*_eq_(C). The dimethylammonium cation was highly disordered and was treated with the SQUEEZE routine (Spek, 2009); the reported crystallographic data takes into account the presence of the cation.

### Crystal data

**Table:**

(C_2_H_8_N)[Cu(C_8_H_4_O_4_)(C_8_H_6_N_3_)]	*Z* = 2
*M**_r_* = 417.91	*F*(000) = 430
Triclinic, *P*1	*D*_x_ = 1.507 Mg m^−^^3^
Hall symbol: -P 1	Mo *K*α radiation, λ = 0.71073 Å
*a* = 8.0978 (16) Å	Cell parameters from 8598 reflections
*b* = 9.7244 (19) Å	θ = 3.0–27.6°
*c* = 11.694 (2) Å	µ = 1.22 mm^−^^1^
α = 89.26 (3)°	*T* = 293 K
β = 89.12 (3)°	Block, blue
γ = 89.64 (3)°	0.24 × 0.22 × 0.21 mm
*V* = 920.7 (3) Å^3^	

### Data collection

**Table:**

Rigaku SCXmini diffractometer	3236 independent reflections
Radiation source: fine-focus sealed tube	2486 reflections with *I* > 2σ(*I*)
Graphite monochromator	*R*_int_ = 0.044
ω scans	θ_max_ = 25.0°, θ_min_ = 3.0°
Absorption correction: multi-scan (*ABSCOR*; Higashi, 1995)	*h* = −9→9
*T*_min_ = 0.759, *T*_max_ = 0.784	*k* = −11→11
8051 measured reflections	*l* = −13→13

### Refinement

**Table:**

Refinement on *F*^2^	Primary atom site location: structure-invariant direct methods
Least-squares matrix: full	Secondary atom site location: difference Fourier map
*R*[*F*^2^ > 2σ(*F*^2^)] = 0.046	Hydrogen site location: inferred from neighbouring sites
*wR*(*F*^2^) = 0.124	H-atom parameters constrained
*S* = 1.06	*w* = 1/[σ^2^(*F*_o_^2^) + (0.0557*P*)^2^ + 0.9233*P*] where *P* = (*F*_o_^2^ + 2*F*_c_^2^)/3
3236 reflections	(Δ/σ)_max_ < 0.001
220 parameters	Δρ_max_ = 0.50 e Å^−^^3^
0 restraints	Δρ_min_ = −0.30 e Å^−^^3^

### Special details

**Table:**

Geometry. All e.s.d.'s (except the e.s.d. in the dihedral angle between two l.s. planes) are estimated using the full covariance matrix. The cell e.s.d.'s are taken into account individually in the estimation of e.s.d.'s in distances, angles and torsion angles; correlations between e.s.d.'s in cell parameters are only used when they are defined by crystal symmetry. An approximate (isotropic) treatment of cell e.s.d.'s is used for estimating e.s.d.'s involving l.s. planes.
Refinement. Refinement of *F*^2^ against ALL reflections. The weighted *R*-factor *wR* and goodness of fit *S* are based on *F*^2^, conventional *R*-factors *R* are based on *F*, with *F* set to zero for negative *F*^2^. The threshold expression of *F*^2^ > σ(*F*^2^) is used only for calculating *R*-factors(gt) *etc*. and is not relevant to the choice of reflections for refinement. *R*-factors based on *F*^2^ are statistically about twice as large as those based on *F*, and *R*- factors based on ALL data will be even larger.

### Fractional atomic coordinates and isotropic or equivalent isotropic displacement parameters (Å^2^)

**Table:**

	*x*	*y*	*z*	*U*_iso_*/*U*_eq_	
Cu1	1.0000	0.5000	0.5000	0.0276 (2)	
Cu2	1.0000	0.5000	0.0000	0.0269 (2)	
O1	0.9981 (3)	0.6272 (3)	0.3683 (2)	0.0309 (6)	
O2	0.8655 (4)	0.7485 (3)	0.4981 (3)	0.0519 (9)	
O3	0.8239 (3)	0.5948 (3)	0.1664 (2)	0.0329 (6)	
O4	1.0087 (3)	0.6914 (3)	0.0509 (2)	0.0318 (6)	
N1	0.1845 (4)	0.4575 (3)	0.1055 (3)	0.0304 (8)	
N2	0.7841 (4)	0.4230 (3)	0.4546 (3)	0.0310 (8)	
N3	0.7012 (4)	0.4575 (3)	0.3568 (3)	0.0284 (7)	
C1	0.9180 (5)	0.7341 (4)	0.3997 (3)	0.0320 (9)	
C2	0.8933 (5)	0.8487 (4)	0.3141 (3)	0.0299 (9)	
C3	0.8962 (5)	0.8307 (4)	0.1954 (3)	0.0287 (9)	
C4	0.9107 (5)	0.6958 (4)	0.1364 (3)	0.0270 (8)	
C5	0.8805 (5)	0.9467 (4)	0.1244 (4)	0.0387 (10)	
H5	0.8842	0.9362	0.0455	0.046*	
C6	0.8600 (6)	1.0754 (5)	0.1688 (4)	0.0504 (12)	
H6	0.8488	1.1511	0.1200	0.061*	
C7	0.8559 (7)	1.0929 (5)	0.2847 (4)	0.0539 (13)	
H7	0.8424	1.1803	0.3150	0.065*	
C8	0.8719 (6)	0.9800 (5)	0.3561 (4)	0.0445 (11)	
H8	0.8683	0.9924	0.4349	0.053*	
C9	0.6856 (5)	0.3392 (4)	0.5137 (3)	0.0349 (10)	
H9	0.7116	0.2992	0.5839	0.042*	
C10	0.5388 (5)	0.3190 (4)	0.4571 (3)	0.0357 (10)	
H10	0.4502	0.2649	0.4812	0.043*	
C11	0.5517 (5)	0.3955 (4)	0.3579 (3)	0.0287 (9)	
C12	0.4293 (4)	0.4167 (4)	0.2673 (3)	0.0283 (9)	
C13	0.2849 (5)	0.3433 (5)	0.2711 (4)	0.0451 (12)	
H13	0.2666	0.2781	0.3287	0.054*	
C14	0.1682 (5)	0.3670 (5)	0.1896 (4)	0.0447 (12)	
H14	0.0715	0.3160	0.1939	0.054*	
C15	0.3227 (6)	0.5277 (6)	0.1017 (4)	0.0567 (14)	
H15	0.3367	0.5935	0.0440	0.068*	
C16	0.4478 (6)	0.5087 (5)	0.1790 (4)	0.0566 (15)	
H16	0.5450	0.5587	0.1710	0.068*	

### Atomic displacement parameters (Å^2^)

**Table:**

	*U*^11^	*U*^22^	*U*^33^	*U*^12^	*U*^13^	*U*^23^
Cu1	0.0263 (4)	0.0308 (4)	0.0260 (4)	−0.0010 (3)	−0.0127 (3)	0.0035 (3)
Cu2	0.0247 (4)	0.0338 (4)	0.0226 (3)	−0.0005 (3)	−0.0098 (3)	−0.0011 (3)
O1	0.0306 (14)	0.0326 (16)	0.0298 (14)	0.0011 (12)	−0.0102 (12)	0.0046 (12)
O2	0.068 (2)	0.055 (2)	0.0314 (17)	0.0063 (17)	0.0068 (16)	0.0069 (15)
O3	0.0355 (15)	0.0350 (16)	0.0283 (14)	−0.0081 (12)	−0.0066 (12)	0.0024 (12)
O4	0.0318 (15)	0.0367 (16)	0.0271 (14)	−0.0033 (12)	−0.0019 (12)	−0.0018 (12)
N1	0.0264 (17)	0.038 (2)	0.0272 (17)	−0.0013 (14)	−0.0066 (14)	0.0004 (15)
N2	0.0301 (18)	0.037 (2)	0.0261 (17)	−0.0010 (15)	−0.0137 (14)	0.0061 (15)
N3	0.0259 (17)	0.0328 (19)	0.0269 (17)	0.0009 (14)	−0.0125 (14)	0.0000 (14)
C1	0.033 (2)	0.037 (2)	0.027 (2)	−0.0043 (18)	−0.0089 (18)	0.0011 (18)
C2	0.033 (2)	0.029 (2)	0.028 (2)	0.0001 (17)	−0.0050 (17)	0.0006 (17)
C3	0.027 (2)	0.032 (2)	0.027 (2)	0.0014 (16)	−0.0038 (16)	0.0019 (17)
C4	0.0250 (19)	0.032 (2)	0.024 (2)	0.0002 (17)	−0.0112 (17)	0.0000 (16)
C5	0.045 (3)	0.042 (3)	0.029 (2)	0.001 (2)	−0.0041 (19)	0.0073 (19)
C6	0.070 (3)	0.029 (3)	0.052 (3)	0.006 (2)	0.000 (3)	0.010 (2)
C7	0.081 (4)	0.027 (2)	0.054 (3)	0.007 (2)	−0.001 (3)	−0.004 (2)
C8	0.059 (3)	0.041 (3)	0.033 (2)	0.000 (2)	−0.005 (2)	−0.006 (2)
C9	0.036 (2)	0.041 (3)	0.028 (2)	−0.0007 (19)	−0.0116 (18)	0.0113 (18)
C10	0.027 (2)	0.047 (3)	0.033 (2)	−0.0069 (18)	−0.0058 (18)	0.0040 (19)
C11	0.024 (2)	0.032 (2)	0.030 (2)	−0.0009 (16)	−0.0063 (17)	−0.0014 (17)
C12	0.024 (2)	0.036 (2)	0.0245 (19)	−0.0001 (16)	−0.0068 (16)	−0.0030 (17)
C13	0.039 (3)	0.062 (3)	0.034 (2)	−0.014 (2)	−0.011 (2)	0.019 (2)
C14	0.031 (2)	0.062 (3)	0.041 (3)	−0.016 (2)	−0.013 (2)	0.015 (2)
C15	0.038 (3)	0.076 (4)	0.056 (3)	−0.016 (2)	−0.024 (2)	0.036 (3)
C16	0.033 (2)	0.073 (4)	0.064 (3)	−0.025 (2)	−0.027 (2)	0.036 (3)

### Geometric parameters (Å, º)

**Table:**

Cu1—O1	1.963 (3)	C3—C4	1.494 (5)
Cu1—O1^i^	1.963 (3)	C5—C6	1.370 (6)
Cu1—N2^i^	1.989 (3)	C5—H5	0.9300
Cu1—N2	1.989 (3)	C6—C7	1.368 (7)
Cu2—O4	1.964 (3)	C6—H6	0.9300
Cu2—O4^ii^	1.964 (3)	C7—C8	1.378 (6)
Cu2—N1^iii^	1.990 (3)	C7—H7	0.9300
Cu2—N1^iv^	1.990 (3)	C8—H8	0.9300
O1—C1	1.276 (5)	C9—C10	1.387 (6)
O2—C1	1.230 (5)	C9—H9	0.9300
O3—C4	1.254 (4)	C10—C11	1.373 (5)
O4—C4	1.268 (4)	C10—H10	0.9300
N1—C15	1.315 (5)	C11—C12	1.474 (5)
N1—C14	1.317 (5)	C12—C16	1.365 (6)
N1—Cu2^v^	1.990 (3)	C12—C13	1.373 (6)
N2—C9	1.324 (5)	C13—C14	1.369 (6)
N2—N3	1.372 (4)	C13—H13	0.9300
N3—C11	1.355 (5)	C14—H14	0.9300
C1—C2	1.503 (5)	C15—C16	1.378 (6)
C2—C8	1.384 (6)	C15—H15	0.9300
C2—C3	1.400 (5)	C16—H16	0.9300
C3—C5	1.399 (5)		
			
O1—Cu1—O1^i^	180.000 (1)	C3—C5—H5	119.3
O1—Cu1—N2^i^	89.29 (12)	C7—C6—C5	120.1 (4)
O1^i^—Cu1—N2^i^	90.71 (12)	C7—C6—H6	119.9
O1—Cu1—N2	90.71 (12)	C5—C6—H6	119.9
O1^i^—Cu1—N2	89.29 (12)	C6—C7—C8	119.4 (4)
N2^i^—Cu1—N2	180.00 (17)	C6—C7—H7	120.3
O4—Cu2—O4^ii^	180.00 (14)	C8—C7—H7	120.3
O4—Cu2—N1^iii^	88.12 (12)	C7—C8—C2	121.9 (4)
O4^ii^—Cu2—N1^iii^	91.88 (12)	C7—C8—H8	119.1
O4—Cu2—N1^iv^	91.88 (12)	C2—C8—H8	119.1
O4^ii^—Cu2—N1^iv^	88.12 (12)	N2—C9—C10	111.0 (3)
N1^iii^—Cu2—N1^iv^	180.00 (19)	N2—C9—H9	124.5
C1—O1—Cu1	106.8 (2)	C10—C9—H9	124.5
C4—O4—Cu2	104.6 (2)	C11—C10—C9	105.5 (3)
C15—N1—C14	116.5 (3)	C11—C10—H10	127.3
C15—N1—Cu2^v^	121.6 (3)	C9—C10—H10	127.3
C14—N1—Cu2^v^	121.6 (3)	N3—C11—C10	107.7 (3)
C9—N2—N3	106.2 (3)	N3—C11—C12	123.1 (3)
C9—N2—Cu1	128.4 (3)	C10—C11—C12	129.1 (3)
N3—N2—Cu1	125.2 (2)	C16—C12—C13	116.6 (4)
C11—N3—N2	109.6 (3)	C16—C12—C11	123.9 (3)
O2—C1—O1	122.5 (4)	C13—C12—C11	119.5 (3)
O2—C1—C2	119.0 (4)	C14—C13—C12	119.5 (4)
O1—C1—C2	118.5 (3)	C14—C13—H13	120.2
C8—C2—C3	118.7 (4)	C12—C13—H13	120.2
C8—C2—C1	117.3 (4)	N1—C14—C13	124.0 (4)
C3—C2—C1	124.0 (3)	N1—C14—H14	118.0
C5—C3—C2	118.6 (4)	C13—C14—H14	118.0
C5—C3—C4	116.0 (3)	N1—C15—C16	123.3 (4)
C2—C3—C4	125.4 (3)	N1—C15—H15	118.4
O3—C4—O4	122.1 (4)	C16—C15—H15	118.4
O3—C4—C3	121.5 (3)	C12—C16—C15	120.1 (4)
O4—C4—C3	116.3 (3)	C12—C16—H16	120.0
C6—C5—C3	121.3 (4)	C15—C16—H16	120.0
C6—C5—H5	119.3		

Symmetry codes: (i) −*x*+2, −*y*+1, −*z*+1; (ii) −*x*+2, −*y*+1, −*z*; (iii) *x*+1, *y*, *z*; (iv) −*x*+1, −*y*+1, −*z*; (v) *x*−1, *y*, *z*.

### Hydrogen-bond geometry (Å, º)

Cg1 is the centroid of the N2,N3,C9–C11 ring.

**Table:**

*D*—H···*A*	*D*—H	H···*A*	*D*···*A*	*D*—H···*A*
C7—H7···*Cg*1^vi^	0.93	2.85	3.698 (5)	152

Symmetry code: (vi) *x*, *y*+1, *z*.

## References

[bb1] Brandenburg, K. (1999). *DIAMOND* Crystal Impact GbR, Bonn, Germany.

[bb2] Davies, G. M., Adams, H. & Ward, M. D. (2005). *Acta Cryst.* C**61**, m485–m487. 10.1107/S0108270105031732 16272588

[bb3] Guo, J.-H. (2010). *Acta Cryst.* E**66**, m1206. 10.1107/S1600536810034616 PMC2983402 21587365

[bb4] Higashi, T. (1995). *ABSCOR* Rigaku Corporation, Tokyo, Japan.

[bb5] Johnson, C. K. (1976). *ORTEPII* Report ORNL-5138, Oak Ridge National Laboratory, Tennessee, USA.

[bb6] Rigaku (2005). *CrystalClear* Rigaku Corporation, Tokyo, Japan.

[bb7] Sheldrick, G. M. (2008). *Acta Cryst.* A**64**, 112–122. 10.1107/S0108767307043930 18156677

[bb8] Spek, A. L. (2009). *Acta Cryst.* D**65**, 148–155. 10.1107/S090744490804362X PMC2631630 19171970

[bb9] Tan, Z.-D., Tan, F.-J., Tan, B. & Yi, Z.-W. (2011). *Acta Cryst.* E**67**, m1512.

[bb10] Yan, Y., Yu, W.-J. & Chen, J. (2012). *Acta Cryst.* E**68**, m129–m130. 10.1107/S1600536812000128 PMC3274868 22346815

